# Supplementary material for: Dietary vitamin E reaches the mitochondria in the flight muscle of zebra finches but only if they exercise
Source: PLoS One. 2021 Jun 28;16(6):e0253264. doi: 10.1371/journal.pone.0253264 (PMC8238215; doi:10.1371/journal.pone.0253264)
Supplement: Attachment — Submitted filename: PONE-D-20-29149_reviewer_Comments.pdf [file pone.0253264.s003.pdf]

# PLOS ONE

## Dietary antioxidants reach the mitochondria in the flight muscle of birds but only if they exercise

--Manuscript Draft--

| | |
|---|---|
| **Manuscript Number:** | PONE-D-20-29149 |
| **Article Type:** | Research Article |
| **Full Title:** | Dietary antioxidants reach the mitochondria in the flight muscle of birds but only if they exercise |
| **Short Title:** | Vitamin E is absorbed and transported to bird flight muscles but only if exercising |
| **Corresponding Author:** | Clara Cooper-Mullin<br>University of Rhode Island<br>Kingston, Rhode Island UNITED STATES |
| **Keywords:** | Vitamin E; zebra finch; deuterium; α-tocopherol; bioavailability; oxidative stress |
| **Abstract:** | Whether dietary antioxidants are effective for alleviating oxidative costs associated with energy-demanding life events first requires they are successfully absorbed in the digestive tract and transported to sites associated with reactive species production (e.g. the mitochondria). Flying birds are under high energy and oxidative demands, and although birds commonly ingest dietary antioxidants in the wild, the bioavailability of these consumed antioxidants is poorly understood. We show for the first time that an ingested lipophilic antioxidant, α-tocopherol, reached the mitochondria in the flight muscles of a songbird but only if they regularly exercise (60 min of perch-to-perch flights two times in a day or 8.5 km day -1 ). Deuterated α-tocopherol was found in the blood of exercise-trained zebra finches within 6.5 hrs and in isolated mitochondria from pectoral muscle within 22.5 hrs, but never reached the mitochondria in caged sedentary control birds. This rapid pace (within a day) and extent of metabolic routing of a dietary antioxidant to muscle mitochondria means that daily consumption of such dietary sources can help to pay the inevitable oxidative costs of metabolism, but only when combined with regular exercise.8.3.3 |
| **Order of Authors:** | Clara Cooper-Mullin |
| | Wales A. Carter |
| | Ronald S. Amato |
| | David Podlesak |
| | Scott R. McWilliams |
| **Additional Information:** | |
| **Question** | **Response** |
| **Financial Disclosure**<br><br>Enter a financial disclosure statement that describes the sources of funding for the work included in this submission. Review the submission guidelines for detailed requirements. View published research articles from *PLOS ONE* for specific examples.<br><br>This statement is required for submission and **will appear in the published article** if the submission is accepted. Please make sure it is accurate. | SRM: This work was supported by the National Science Foundation [IOS-0748349] https://www.nsf.gov/<br>and the United States Department of Agriculture [RIAES-538748] https://www.usda.gov/. The funders had no role in study design, data collection and analysis, decision to publish, or preparation of the manuscript. |

Powered by Editorial Manager® and ProduXion Manager® from Aries Systems Corporation

| | |
|---|---|
| **Unfunded studies**<br>Enter: *The author(s) received no specific funding for this work.*<br><br>**Funded studies**<br>Enter a statement with the following details:<br>• Initials of the authors who received each award<br>• Grant numbers awarded to each author<br>• The full name of each funder<br>• URL of each funder website<br>• Did the sponsors or funders play any role in the study design, data collection and analysis, decision to publish, or preparation of the manuscript?<br>• **NO** - Include this sentence at the end of your statement: *The funders had no role in study design, data collection and analysis, decision to publish, or preparation of the manuscript.*<br>• **YES** - Specify the role(s) played.<br><br>* typeset | |
| **Competing Interests**<br><br>Use the instructions below to enter a competing interest statement for this submission. On behalf of all authors, disclose any competing interests that could be perceived to bias this work—acknowledging all financial support and any other relevant financial or non-financial competing interests.<br><br>This statement **will appear in the published article** if the submission is accepted. Please make sure it is accurate. View published research articles from *PLOS ONE* for specific examples. | The authors have declared that no competing interests exist. |

| | |
|---|---|
| **NO authors have competing interests**<br><br>Enter: *The authors have declared that no competing interests exist.*<br><br>**Authors with competing interests**<br><br>Enter competing interest details beginning with this statement:<br><br>*I have read the journal's policy and the authors of this manuscript have the following competing interests: [insert competing interests here]*<br><br>* typeset | |
| **Ethics Statement**<br><br>Enter an ethics statement for this submission. This statement is required if the study involved:<br><br>• Human participants<br>• Human specimens or tissue<br>• Vertebrate animals or cephalopods<br>• Vertebrate embryos or tissues<br>• Field research<br><br>Write "N/A" if the submission does not require an ethics statement.<br><br>General guidance is provided below. Consult the submission guidelines for detailed instructions. **Make sure that all information entered here is included in the Methods section of the manuscript.** | All care and experimental procedures were reviewed and approved by the University of Rhode Island’s Institutional Animal<br>Care and Use Committee under protocol AN11-12-009. Birds were sacrificed by cervical dislocation. |

Powered by Editorial Manager® and ProduXion Manager® from Aries Systems Corporation

| | |
|---|---|
| **Format for specific study types**<br><br>**Human Subject Research (involving human participants and/or tissue)**<br>• Give the name of the institutional review board or ethics committee that approved the study<br>• Include the approval number and/or a statement indicating approval of this research<br>• Indicate the form of consent obtained (written/oral) or the reason that consent was not obtained (e.g. the data were analyzed anonymously)<br><br>**Animal Research (involving vertebrate animals, embryos or tissues)**<br>• Provide the name of the Institutional Animal Care and Use Committee (IACUC) or other relevant ethics board that reviewed the study protocol, and indicate whether they approved this research or granted a formal waiver of ethical approval<br>• Include an approval number if one was obtained<br>• If the study involved *non-human primates*, add *additional details* about animal welfare and steps taken to ameliorate suffering<br>• If anesthesia, euthanasia, or any kind of animal sacrifice is part of the study, include briefly which substances and/or methods were applied<br><br>**Field Research**<br><br>Include the following details if this study involves the collection of plant, animal, or other materials from a natural setting:<br>• Field permit number<br>• Name of the institution or relevant body that granted permission | |
| **Data Availability**<br><br>Authors are required to make all data underlying the findings described fully available, without restriction, and from the time of publication. PLOS allows rare exceptions to address legal and ethical concerns. See the PLOS Data Policy and FAQ for detailed information. | Yes - all data are fully available without restriction |

| | |
|---|---|
| A Data Availability Statement describing where the data can be found is required at submission. Your answers to this question constitute the Data Availability Statement and **will be published in the article**, if accepted.<br><br>**Important:** Stating 'data available on request from the author' is not sufficient. If your data are only available upon request, select 'No' for the first question and explain your exceptional situation in the text box.<br><br>Do the authors confirm that all data underlying the findings described in their manuscript are fully available without restriction? | |
| **Describe where the data may be found in full sentences. If you are copying our sample text, replace any instances of XXX with the appropriate details.**<br><br>• If the data are **held or will be held in a public repository**, include URLs, accession numbers or DOIs. If this information will only be available after acceptance, indicate this by ticking the box below. For example: *All XXX files are available from the XXX database (accession number(s) XXX, XXX.).*<br>• If the data are all contained **within the manuscript and/or Supporting Information files**, enter the following: *All relevant data are within the manuscript and its Supporting Information files.*<br>• If neither of these applies but you are able to provide **details of access elsewhere**, with or without limitations, please do so. For example:<br><br>*Data cannot be shared publicly because of [XXX]. Data are available from the XXX Institutional Data Access / Ethics Committee (contact via XXX) for researchers who meet the criteria for access to confidential data.*<br><br>*The data underlying the results presented in the study are available from (include the name of the third party* | All relevant data are within the manuscript and its Supporting Information files. Data and R Code used to create the figures and carry out the analysis are currently included as supplementary files, but upon acceptance will be moved to a public repository (Dryad). |

| | |
|---|---|
| *and contact information or URL).*<br>• This text is appropriate if the data are owned by a third party and authors do not have permission to share the data.<br><br>* typeset | |
| Additional data availability information: | Tick here if the URLs/accession numbers/DOIs will be available only after acceptance of the manuscript for publication so that we can ensure their inclusion before publication. |

Powered by Editorial Manager® and ProduXion Manager® from Aries Systems Corporation

Manuscript

Click here to access/download;Manuscript;ZEBE_PlosOne_submit.docx

**Full Title: Dietary antioxidants reach the mitochondria in the flight muscle of birds but only if they exercise**

**Short Title: Vitamin E is absorbed and transported to bird flight muscles but only if exercising**

**Clara Cooper-Mullin[¶1], Wales A. Carter[1], Ronald S. Amato[2], David Podlesak[2], and Scott R. McWilliams[¶1]**

[1]Department of Natural Resources Science, University of Rhode Island, Kingston Rhode Island, United States of America

[2]Los Alamos National Laboratory, Los Alamos, New Mexico, United States of America

[*]Corresponding Author:

Email: ccooper-mullin@uri.edu (CC-M)

[¶]These authors contributed equally to this work.

**Keywords**

Vitamin E; zebra finch; deuterium; α-tocopherol; bioavailability; oxidative stress

# Abstract

Whether dietary antioxidants are effective for alleviating oxidative costs associated with energy-demanding life events first requires they are successfully absorbed in the digestive tract and transported to sites associated with reactive species production (e.g. the mitochondria). Flying birds are under high energy and oxidative demands, and although birds commonly ingest dietary antioxidants in the wild, the bioavailability of these consumed antioxidants is poorly understood. We show for the first time that an ingested lipophilic antioxidant, α-tocopherol, reached the mitochondria in the flight muscles of a songbird but only if they regularly exercise (60 min of perch-to-perch flights two times in a day or 8.5 km day$^{-1}$). Deuterated α-tocopherol was found in the blood of exercise-trained zebra finches within 6.5 hrs and in isolated mitochondria from pectoral muscle within 22.5 hrs, but never reached the mitochondria in caged sedentary control birds. This rapid pace (within a day) and extent of metabolic routing of a dietary antioxidant to muscle mitochondria means that daily consumption of such dietary sources can help to pay the inevitable oxidative costs of metabolism, but only when combined with regular exercise.


# Introduction

All animals must produce energy to fuel metabolism and an inevitable byproduct of this metabolism are reactive species (RS) produced in the mitochondria that must be quenched to avoid oxidative damage to mitochondria, cells, and tissues [1–7]. Although animals have a complex endogenous antioxidant system that can and does respond to oxidative challenges, upregulating this endogenous system costs energy [8–10]. Consumption of dietary antioxidants (e.g. tocopherols, polyphenols) can mitigate this cost [9] and can play an essential role in preventing oxidative damage - especially during energy-demanding life events such as growth,

exercise (e.g. migration), breeding, or when faced with an immune challenge [11–15]. Supplementing the diets of athletes (humans, birds, or mammals) with certain antioxidants prior to high-intensity exercise training protects against oxidative damage to lipids and muscles [16–19]. A wide variety of animals prefer foods with certain antioxidants [20–22] and these micronutrients are abundant in many foods consumed by wild animals [9,11,20–26]. Although the functional importance of dietary antioxidants to animal health and performance is recognized [16,27,28], no previous studies have demonstrated that consumed antioxidants are transported to the primary site of reactive species production, the mitochondria, and whether this depends at all on the physiological state of the animal.

Flying birds offer a particularly relevant system for investigating how exercise affects the bioavailability and efficacy of dietary antioxidants, given the high energetic costs of flight [29–31], birds reliance on fats as fuel [32–37], and their preference for foods rich in antioxidants [20,22,23,38–40]. Flight is a particularly energetically expensive form of exercise and is primarily fueled by catabolizing stored fat - nature's most energy dense, yet oxidatively vulnerable fuel [2,41,42]. Due to the nature of aerodynamics, perch-to-perch flights like the flight performed while foraging increase an individual's metabolic rate up to 30 times basal metabolic rate (BMR) [31,43,44] and, therefore, likely result in a high oxidative load [2]. Likewise, long-duration, sustained flights by migrating birds, some of the world's most incredible athletes, require prolonged periods operating at high metabolic rates (>10X BMR) while not eating or drinking [32,45–49]. Birds regularly consume lipophilic and hydrophilic antioxidants from fruits during fall migration [20,22,38,50], but evidence that these antioxidants protect against damage by RS during high-intensity exercise is murky due in part to our inability to directly measure rates of RS production *in vivo* [2,51–53], as well as the complexity of the

antioxidant system [2,7,51,54–56]. Importantly, if and only if dietary antioxidants are absorbed and transported to the mitochondria, can they effectively alleviate the oxidative costs associated with energy-demanding life events such as flying.

α-Tocopherol, one of eight stereoisomers of vitamin E, is an essential dietary antioxidant (i.e., cannot be produced de novo by any vertebrate and so must be consumed) that acts as a chain-breaker in the propagation of lipid peroxidation [1,57,58], and once oxidized can be recycled by vitamin C *in vivo* [59]. The livestock and poultry industries have long acknowledged vitamin E to be crucial for preventing oxidation of meat, and have focused on understanding how to use dietary supplementation to increase subcellular concentrations of vitamin E [60,61]. Holstein and beef steers that consumed vitamin E-supplemented diets had higher tocopherol concentrations in muscle mitochondrial fractions as compared to steers not given dietary vitamin E [62] although this has not been shown in wild mammals or birds. Additionally, dietary supplementation increased α-tocopherol content of the subcellular membranes of various porcine and chicken muscles [63,64]. Although mitochondria are known to have high α-tocopherol concentrations, especially in the inner mitochondrial membrane [65,66], the timing and deposition of α-tocopherol from the diet to plasma to mitochondria in different tissues remains largely unknown. Further, as α-tocopherol can be recycled [59], it is unclear whether an observed increase in α-tocopherol concentrations in subcellular fractions are a direct result of deposition from the diet or an indirect effect on the recycling of α-tocopherol by ascorbic acid.

To our knowledge, no previous studies have directly demonstrated (a) that α-tocopherol from the diet is metabolically routed to the muscle mitochondria in a volant species with relatively higher metabolic rates and potential reactive species production, and (b) how the metabolic routing is directly affected by very high-energy demanding exercise, in this case in

actively-flying birds [45,67,68]. This is the first study in passerines to trace vitamin E from the diet into circulation and then mitochondria of the primary flight muscle, and the first in birds to examine whether exercise changes the absorption and deposition of vitamin E into tissues and organelles. Given the high energy and oxidative costs of flying in birds, elucidating the pace and extent of metabolic routing of a commonly consumed dietary antioxidant would reveal the efficacy of such dietary sources for paying these oxidative costs. Specifically, we tested the following hypotheses: (1) Lipid-soluble dietary antioxidants are routed to the mitochondria within 2-30 hours of ingestion in agreement with previous studies on the appearance and half-life of vitamin E in plasma of livestock and humans [61,69,70] (2) The pace of this routing of dietary antioxidants depends on exercise with faster routing in flown vs. sedentary birds. We also provide the first estimates of the time course of metabolism of lipid-soluble dietary antioxidants in sedentary and flying birds.

# Methods

## Exercise Training:

We performed this experiment on zebra finches obtained from a known-age captive population of birds at Sacred Heart University [54,71]. This captive population allowed us more control over potential variation in age [72,73], sex [74,75], gastrointestinal microbiome composition [76,77], and diet [1,78,79], which can influence absorption of dietary antioxidants or the response of the antioxidant system to exercise. Importantly, zebra finches are passerines with similar genetic antioxidant regulatory pathways to wild migratory songbirds [80]. Zebra finches are also social and easily trained to fly in large numbers in a flight arena, and we were

able to avoid sacrificing wild migratory songbirds with potentially declining population sizes [81].

The birds used in this experiment were part of a larger experiment focused on lipid turnover rates and changes in the antioxidant system in exercised and non-exercised birds [54,71]. The finches (n = 65) were randomly assigned to an exercise-trained treatment group (N = 33, 16 males, 17 females) or an untrained group (N = 32, 15 males, 17 females). Prior to exercise training, the 65 zebra finches were kept in same-sex aviaries (2.1 x 0.9 x 1.8 m = L x W x H) for 8 weeks on a 14 h:10 h light:dark cycle under full-spectrum light, with lights on at 06:00. Birds were banded with an aluminum numbered band on their right leg for individual recognition with one color band on the left leg to indicate treatment (trained males = red; untrained males = green). During those 8 weeks, birds acclimated to the aviaries, and to a standard mixed-seed diet (Abbaseed #3700, Hillside, NJ USA) composed mainly of canarygrass (*Phalaris canariensis*) and had *ad libitum* access to water, grit, and cuttlebone. All zebra finches were given fresh kale, a source of ascorbic acid and α-tocopherol, once weekly [54,71,82].

The exercise-trained zebra finches were subjected daily to two one-hour periods of stop-and-go perch-to-perch flights (11:00 – 12:00 and 13:30 – 14:30) in a 6 (*l*) x 3 (*w*) x 2 m tall flight arena for up to 70 days (see [54,71,79,83,84]. Each day during the 48 or 70 days of exercise training, zebra finches flew between perches located 4 m apart in opposite corners of the arena. A cloth wall partially divided the arena in the middle such that birds departing from a given perch flew to one side of the arena and returned on the opposite side. During the two 1 h sessions each day, a handler walked clockwise around the arena for 300 laps per hour, which resulted over the two hrs in about 1200 8-m flights or approximately 8.5 km per day. This type of short-burst flight incurs energetic costs that are approximately 3x higher than sustained flight [31].

During the two 1 h exercise periods, we removed food and water from the cages housing untrained birds to ensure both treatment (exercise-trained) and control (untrained) birds were similarly fasted. Although untrained birds were able to move around unhindered within their cages, they were never able to fly more than 2.1 m at a time and occasional observations of birds in the cages suggest that they spent most of their time perched and not flying.

### Blood Sampling, Mitochondrial Isolation and Mass Spectrometry:

After 48 days of daily exercise training, we randomly selected 8 male zebra finches from the untrained group and 4 male zebra finches from the trained group to be gavaged with 150 μL of deuterated α – tocopherol ($d_6$-RRR- α – tocopherol acetate, a gift from Dr. John Lodge, St. Thomas' Hospital, London) dissolved in olive oil. The gavaged amount of deuterated α – tocopherol (30 IU/dose) was chosen based on recommended supplements for poultry and to avoid any negative effects of over supplementation [19,85]. After 70 days of exercise training, we randomly selected a second group of 3 male zebra finches from the trained group to be gavaged with 150 μL of deuterated α – tocopherol ($\delta$D α-tocopherol), and 3 male zebra finches from the untrained group to be gavaged with 150 μL of olive oil. This latter group was used to determine the natural background level of deuterated hydrogen in the blood and isolated mitochondria of zebra finches and is generally assumed to be highly stable [86,87]. To determine a timecourse for incorporation of $\delta$D α-tocopherol into blood and mitochondria, we randomly selected whether birds were gavaged the night before (22.5 hours prior to sampling) or in the morning (6.5 hours prior to sampling). Directly after the second one-hr flight training, we sacrificed the 8 trained and paired untrained birds, took a blood sample from the severed carotid artery, and removed the right pectoral muscle. Blood samples were flash frozen in liquid nitrogen and stored at 80$^{o}$C until further analysis. Pectoral muscle was placed in an ice-cold high sucrose

isolation buffer solution modified from Scott et al. [88]. Mitochondria from each bird was isolated using differential centrifugation and a Percoll gradient [88–91]. Both mitochondria and blood samples were freeze dried and run on a mass spectrometer to detect amount of deuterated α – tocopherol.

**Sample Preparation:**

Blood samples ($n = 18$), isolated mitochondria ($n = 17$) and two muscle samples were freeze dried at the University of Rhode Island and mailed to Los Alamos National Laboratories for further analysis. We used the two muscle samples to establish appropriate sample aliquot sizes for analysis of the blood and isolated mitochondria samples and determined that 150- μg tissue yielded sufficient signal for analysis. One of these two muscle samples was obtained from a bird gavaged with δD-α-tocopherol in olive oil and the other from a bird gavaged with only olive oil. Aliquots of each muscle sample in 15-ml glass vials (uncapped) equilibrated with the local air for approximately 4 days to allow hydrogen isotopic exchange prior to analysis. Upon arrival in New Mexico, blood and mitochondria samples were also allowed to equilibrate with local air and freeze dried again for 24 hours on non-consecutive days (48 hours total). We weighed samples in duplicate into silver foil capsules before a second round of drying in a vacuum desiccator for approximately one week.

**Isotope Ratio Mass Spectrometry:**

We ran samples in an Isotope Ratio Mass Spectrometry (IRMS) system, consisting of a Delta Plus IRMS (Thermo Scientific, Bremen, GE), high temperature thermal conversion (Thermo Scientific TC/EA) and Conflo IV Interface (Thermo Scientific). Samples in silver foil capsules were dropped via autosampler into a heated glassy carbon column at 1400 °C to convert organic and inorganic constituents into $H_2$¬, $N_2$, and CO gases. All gases were swept into an isothermally packed gas chromatography (GC) column with an ultra-pure helium carrier gas and

separated. The carrier plus separated sample gases were passed to the Conflo IV interface which controls the amount of sample and reference gas sent to the spectrometer. We analyzed a reference gas (ultra-pure hydrogen) twice prior to each sample analysis and compared the sample isotopic ratios to the reference gas for ratio determination expressed as a delta value by Isodat 3.0 (Thermo) software. The IRMS measured ions at mass-to-charge (m/z) ratios of 2 ($^{1}H_2$) and 3 ($^{1}H_2H$). Additionally, we analyzed several reference materials of certified isotopic composition at the beginning, end and periodically throughout the analysis. Well characterized working standards (USGS-42 Tibetan Human Hair, and USGS-43 Indian Human Hair) were simultaneously analyzed and scaled to accepted international standards (VSMOW, Vienna Standard Mean Ocean Water). Post analysis delta values were normalized to these standards. Therefore, sample delta values are a delta scale calibrated to the VSMOW standard of $^{2}H/^{1}H$ = 0.00 ± 0.3. All samples were run in triplicate. Final sample sizes varied after analysis as some samples were not able to be dried completely (Blood Samples: n = 3 Background, 7 Trained, 8 Untrained; Mitochondria Samples: n = 3 Background, 7 Trained, 7 Untrained). Background mitochondria deuterium values (range: -53.29 to -45.01) and background blood deuterium values (range: -134.03 to -130.17) were stable with little variation.

## Statistics:

We used separate linear models for whole blood and mitochondria to determine if the amount of $\delta$D-α-tocopherol in a tissue varied with treatment group (Trained, Untrained) as referenced by background deuterium levels for each tissue, the time elapsed after a bird was gavaged, and the amount of administered δD α-tocopherol. We used the information-theoretic approach based on Akaike's Information Criterion for small sample sizes ($\Delta$ AICc < 2) and Akaike weights (wi) to select the best model for each tissue [92]. We report the results of the

best model for each tissue (Table 1). We visually inspected residual plots and did not reveal any noticeable deviations from homoscedasticity or normality.

We estimated mean retention time of $\delta$D-α-tocopherol in whole blood (τ) using an exponential decay function:

$$y_t = y_\infty + ae^{(-\frac{t}{\tau})} \tag{1}$$

where $y_t$ is the measured $\delta$D value of blood at time t, $y_\infty$ is the estimated asymptotic, or background, $\delta$D value of the blood, *a* is the estimated difference between the $\delta$D values of blood sampled 6.5 hrs after gavage and at equilibrium with background $\delta$D samples, *t* is the measured time since gavage in hours, and τ is the estimated mean retention time of $\delta$D-α-tocopherol. Since the time at which $\delta$D in blood would return to background was unknown, we fit the model using a range of specified time values for the asymptote (30 to 250 hours) until the model converged, and then estimated τ from the model with the best fit. All statistics were performed with R Core Team version 3.6.3 (2020).

# Results

Whole blood δD-α-tocopherol was higher than background for exercise-trained zebra finches at both 6.5 hrs and 22.5 hrs, but no time points were significantly different from background for untrained zebra finches (Fig 1, $R^2$ = 0.79, Trained: ß = 93.45, $T_{3,14}$ = 5.03, $P < 0.001$; Untrained: ß = 8.59, $T_{3,14}$ = 0.46, $P$ = 0.65; Hours Since Gavage: ß = -1.55, $T_{3,14}$ = -2.18, $P$ = 0.05). Deuterated α-tocopherol appeared in mitochondria isolated from pectoral muscle of exercise-trained birds within 22.5 hrs, but δD-α-tocopherol was never higher than baseline in mitochondria isolated from untrained birds (Fig 2, $R^2$ = 0.70, Interaction: ß = 1.80, $T_{3,10}$ = 3.65, $P$ = 0.004 ; Trained: ß = -12.73, $T_{3,10}$ = -1.60, $P$ = 0.15; Hours Since Gavage: ß = -0.28, $T_{3,10}$ = -

0.80, *P* = 0.44). Estimated retention time (τ) in whole blood converged on a value of 24.72 ± 12.76 by 180+ hrs after gavage and the median residence time, or half-life (ln(2) *τ), of gavaged vitamin E in whole blood of exercise-trained zebra finches was estimated at 17.1 hrs. In other words, 90% of gavaged δD-α-tocopherol that appeared in the blood would be replaced or deposited into tissues within 2.4 days.

**Figure 1. Whole blood δD-α-tocopherol values from background (dark blue circle), trained (red) and untrained (light blue) Zebra Finches.**

Only exercise-trained birds had deuterium values that were higher than background, and exercise-trained birds gavaged 6.5 hours prior to sampling (red star) had higher deuterium values than birds gavaged 22.5 hours prior to sampling (red triangle). Deuterium values in the blood of untrained birds were not different from background at either sampling timepoint (gavaged 6.5 hours prior to sampling = light blue star; gavaged 22.5 hours prior to sampling = light blue triangle). Lighter colored points represent the raw data.

**Figure 2. δD-α-tocopherol values of mitochondria isolated from the pectoral muscles of background (dark blue circle), trained (red) and untrained (light blue) Zebra Finches.**

Only exercise-trained birds had deuterium values that were higher than background, and exercise-trained birds gavaged 6.5 hours prior to sampling (red star) had lower deuterium values than birds gavaged 22.5 hours prior to sampling (red triangle). Deuterium values in the blood of untrained birds were not different from background at either sampling timepoint (gavaged 6.5

hours prior to sampling = light blue star; gavaged 22.5 hours prior to sampling = light blue triangle). Lighter colored points represent the raw data.

## Discussion

We show for the first time that an ingested lipophilic antioxidant reached the mitochondria in the flight muscles of a songbird and that uptake and metabolism of this dietary antioxidant depended on exercise. Specifically, δD-α-tocopherol was found in the blood and mitochondria of zebra finches within 6.5 and 22.5 hrs, respectively, but only if the birds were exercise-trained. These results indicate that exercise facilitated the absorption and deposition of vitamin E likely due to a need for external antioxidants to combat increased reactive species production associated with intense exercise [93,94], and/or by inducing muscle cells to actively produce more α-tocopherol rich mitochondria [95–97]. As the most important physiological function of vitamin E is as an antioxidant against free radical-mediated lipid peroxidation [98,99], the first explanation seems likely. The absence of δD-α-tocopherol levels above baseline in untrained birds suggests that any baseline reactive species generation or that associated with short flights within the cages could be neutralized via endogenous antioxidants (enzymes, sacrificial molecules, or stores of dietary antioxidants), or were in low enough of a dose to act solely as cellular messengers rather than cause overwhelming damage [7,100]. It also indicates that birds, like mammals, have mechanisms to maintain plasma α-tocopherol concentration [70,101], and prevent the buildup of excess α-tocopherol by preventing absorption or through efficient metabolism and excretion by the liver.

Although δD-α-tocopherol was detected in whole blood from exercise-trained zebra finches within 6.5 hours after gavage, deposition of α-tocopherol into pectoral muscle mitochondria took more than 6.5 hrs and less than 22.5 hrs.  We estimated that 50% of ingested

vitamin E in whole blood would be replaced within ~17 hrs, and 90% replaced within 2.4 days in exercised finches. This residence time of vitamin E in the blood of trained zebra finches is more rapid than in plasma of humans given deuterium-labeled foods (half-life = ~ 30 hrs), and more rapid than uptake into human erythrocytes [101–103]. Many more such studies of residence time of vitamin E across a broader suite of species and body sizes are required before any conclusions can be made about the allometry of such metabolism [83]. However, the implication for migratory passerines is that vitamin E, available in preferred fruits such as Arrowwood Viburnum (*Viburnum dentatum*) at fall migration stopover sites [22], would be available in their muscle mitochondria within 22.5 hours after ingestion. In other words, vitamin E in fruits eaten by birds today would be available the next night during (nocturnal) migration to defend against reactive species production in their muscle mitochondria.

What remains to be determined is the exact mechanism by which exercise training in small songbirds enables α-tocopherol to be absorbed into the blood and then transported to the mitochondria, although our exercise training regime seems a reasonable approach. We highlight two non-mutually exclusive hypotheses related to such a mechanism: (1) exercise upregulates features of the gut (e.g., digestive enzymes, nutrient transporters, microbial communities) that enhance absorption of α-tocopherol to blood; (2) exercise increases fat metabolism which then facilitates metabolism and transport of dietary antioxidant into muscle mitochondria. Birds rely primarily on fatty acid oxidation to fuel the demands of intense exercise and, in the wild, mechanisms associated with fatty acid oxidation (e.g. activation of PPARs, enzymes such as β-hydroxyacyl-Coenzyme-A dehydrogenase or carnitine palmitoyl transferase) are upregulated prior to seasonal migration [104,105]. Further, migratory flights are fueled by high levels of circulating triglycerides transported by very low density lipoproteins (VLDLs) [30]. Since

vitamin E is lipid soluble and is transported into cells and organelles via VLDLs [78], increased transport of fatty acids and fatty-acyl CoA into muscle mitochondria for β-oxidation likely facilitates the absorption and transport of vitamin E into the mitochondria. Therefore, the ability to benefit from ingested antioxidants where the risk of damage by peroxyl radicals is highest (the mitochondria), could be an inevitable benefit of burning fats as fuel.


## Acknowledgments

We would first like to thank Barbara Pierce for raising the zebra finches used in this experiment, and Dr. John Lodge for providing the deuterated α – tocopherol. We are also grateful for Megan Skrip (and family) who constructed the original flight arena, and for providing guidance for animal care and training throughout the experiment. Thank you to Luke "The Handler" Douglas for his cheerful help while caring for the zebra finches or conducting exercise training. Thank you to Megan Grey and Lara Kazo for their animal care help, Chris Lane for use of his centrifuge, and Alex Gerson for valuable discussions about lipid transport during exercise in birds.

### Funding

This work was supported by the National Science Foundation [IOS-0748349]; and the United States Department of Agriculture [RIAES-538748].


### Authors' Contributions

CCM and SM conceived of the study and designed the study. CCM carried out the captive bird work, blood sampling and mitochondrial isolation, carried out the statistical analysis, and drafted the manuscript; SM provided resources and funding, critically revised the manuscript, and helped with statistical analysis; WC participated in the captive bird work and sampling and provided

edits on the manuscript; RA and DP carried out the deuterium analysis. All authors gave final approval for publication and agree to be held accountable for the work performed therein.

# References


1. Halliwell B, Gutteridge J (2007) Free Radicals in Biology and Medicine. 4th ed. Oxford University Press. 1–704 p.
2. Skrip MM, McWilliams SR (2016) Oxidative balance in birds: An atoms-to-organisms-to-ecology primer for ornithologists. J F Ornithol 87: 1–20. doi:10.1111/jofo.12135.
3. van de Crommenacker J, Hammers M, van der Woude J, Louter M, Santema P, et al. (2017) Oxidative status and fitness components in the Seychelles warbler. Funct Ecol 31: 1210–1219. doi:10.1111/1365-2435.12861.
4. Nikolaidis MG, Kyparos A, Spanou C, Paschalis V, Theodorou AA, et al. (2012) Redox biology of exercise: an integrative and comparative consideration of some overlooked issues. J Exp Biol 215: 1615–1625. doi:10.1242/jeb.067470.
5. Espinosa-Diez C, Miguel V, Mennerich D, Kietzmann T, Sánchez-Pérez P, et al. (2015) Antioxidant responses and cellular adjustments to oxidative stress. Redox Biol 6: 183–197. doi:10.1016/j.redox.2015.07.008.
6. Costantini D, Lindecke O, Pētersons G, Voigt CC (2018) Migratory flight imposes oxidative stress in bats. Curr Zool.
7. Cooper-Mullin C, McWilliams SR (2016) The role of the antioxidant system during intense endurance exercise: lessons from migrating birds. J Exp Biol 219: 3684–3695. doi:10.1242/jeb.123992.
8. Beaulieu M, Schaefer HM (2013) Rethinking the role of dietary antioxidants through the lens of self-medication. Anim Behav 86: 17–24. doi:10.1016/j.anbehav.2013.05.022.

9. Pamplona R, Costantini D (2011) Molecular and structural antioxidant defenses against oxidative stress in animals. Am J Physiol Integr Comp Physiol 301: R843–R863. doi:10.1152/ajpregu.00034.2011.

10. Surai PF (2002) Selenium in poultry nutrition 1. Antioxidant properties, deficiency and toxicity. Worlds Poult Sci J 58: 333–347. doi:10.1079/WPS20020026.

11. Catoni C, Schaefer HM, Peters A (2008) Fruit for health: the effect of flavonoids on humoral immune response and food selection in a frugivorous bird. Funct Ecol 22: 649–654. doi:10.1111/j.1365-2435.2008.01400.x.

12. Costantini D, Meillère A, Carravieri A, Lecomte V, Sorci G, et al. (2014) Oxidative stress in relation to reproduction, contaminants, gender and age in a long-lived seabird. Oecologia 175: 1107–1116. doi:10.1007/s00442-014-2975-x.

13. Sebastiano M, Eens M, Messina S, AbdElgawad H, Pineau K, et al. (2018) Resveratrol supplementation reduces oxidative stress and modulates the immune response in free-living animals during a viral infection. Funct Ecol 32: 2509–2519. doi:10.1111/1365-2435.13195.

14. Larcombe SD, Herborn KA, Alexander L, Arnold KE (2017) Dietary antioxidants in life-history trade-offs: differential effects of a-tocopherol supplementation on blue tit *Cyanistes caeruleus* mothers and offspring during reproduction. Biol J Linn Soc 122: 313–328. doi:10.1093/biolinnean/blx072.

15. Villalba JJ, Costes-Thiré M, Ginane C (2017) Phytochemicals in animal health: Diet selection and trade-offs between costs and benefits. Proc Nutr Soc 76: 113–121. doi:10.1017/S0029665116000719.

16. Watson TA, Callister R, Taylor RD, Sibbritt DW, Macdonald-Wicks LK, et al. (2005)

Antioxidant restriction and oxidative stress in short-duration exhaustive exercise. Med Sci Sports Exerc 37: 63–71. doi:10.1249/01.MSS.0000150016.46508.A1.

17. Rokitzki L, Logemann E, Sagredos AN, Murphy M, Wetzel-Roth W, et al. (1994) Lipid peroxidation and antioxidative vitamins under extreme endurance stress. Acta Physiol Scand 151: 149–158. doi:10.1111/j.1748-1716.1994.tb09732.x.

18. Larcombe SD, Coffey JS, Bann D, Alexander L, Arnold KE (2010) Impacts of dietary antioxidants and flight training on post-exercise oxidative damage in adult parrots. Comp Biochem Physiol - B Biochem Mol Biol 155: 49–53. doi:10.1016/j.cbpb.2009.09.009.

19. Hall ZJ, Bauchinger U, Gerson AR, Price ER, Langlois LA, et al. (2014) Site-specific regulation of adult neurogenesis by dietary fatty acid content, vitamin E and flight exercise in European starlings. Eur J Neurosci 39: 875–882. doi:10.1111/ejn.12456.

20. Alan RR, McWilliams SR, McGraw KJ (2013) The importance of antioxidants for avian fruit selection during autumn migration. Wilson J Ornithol 125: 513–525. doi:10.1676/13-014.1.

21. Homkes AT, Gable TD, Windels SK, Bump JK (2020) Berry important? Wolf provisions pups with berries in northern Minnesota. Wildl Soc Bull 44: 221–223. doi:10.1002/wsb.1065.

22. Bolser JA, Alan RR, Smith AD, Li L, Seeram NP, et al. (2012) Birds select fruits with more anthocyanins during autumn migration. Wilson J Ornithol 124: 000–000.

23. Schaefer HM, McGraw K, Catoni C (2008) Birds use fruit colour as honest signal of dietary antioxidant rewards. Funct Ecol 22: 303–310. doi:10.1111/j.1365-2435.2007.01363.x.

24. Casagrande S, Pinxten R, Eens M (2016) Honest signaling and oxidative stress: the

special case of avian acoustic communication. Front Ecol Evol 4: 1–10. doi:10.3389/fevo.2016.00052.

25. Bessa J, Sousa C, Hockings KJ (2015) Feeding ecology of chimpanzees (<i>Pan troglodytes verus<\i>) inhabiting a forest-mangrove-savanna-agricultural matrix at Caiquene-Cadique, Cantanhez National Park, Guinea-Bissau. Am J Primatol 77: 651–665. doi:10.1002/ajp.22388.

26. Gable TD, Windels SK, Bruggink JG, Barber-Meyer SM (2018) Weekly summer diet of gray wolves (Canis lupus) in northeastern Minnesota. Am Midl Nat: 15–27.

27. Polyak E, Ostrovsky J, Peng M, Dingley SD, Tsukikawa M, et al. (2018) N-acetylcysteine and vitamin E rescue animal longevity and cellular oxidative stress in pre-clinical models of mitochondrial complex I disease. Mol Genet Metab 123: 449–462. doi:10.1016/j.ymgme.2018.02.013.

28. Marri V, Richner H (2014) Differential effects of vitamins E and C and carotenoids on growth, resistance to oxidative stress, fledging success and plumage colouration in wild great tits. J Exp Biol 217: 1478–1484. doi:10.1242/jeb.096826.

29. Weber TP, Ens BJ, Houston AI (1998) Optimal avian migration: A dynamic model of fuel stores and site use. Evol Ecol 12: 377–401. doi:10.1023/A:1006560420310.

30. Jenni L, Jenni-Eiermann SS (1998) Fuel supply and metabolic constraints in migrating birds. J Avian Biol 29: 521–528. doi:10.1016/S0305-0491(00)80104-2.

31. Nudds RL, Bryant DM (2000) The energetic cost of short flights in birds. J Exp Biol 203: 1561–1572.

32. Gerson AR, Guglielmo CG (2013) Energetics and metabolite profiles during early flight in American robins (*Turdus Migratorius*). J Comp Physiol B Biochem Syst Environ

Physiol 183: 983–991. doi:10.1007/s00360-013-0767-y.

33. Jenni-Eiermann SS, Jenni LL, Kvist AA, Lindström AA, Piersma TT, et al. (2002) Fuel use and metabolic response to endurance exercise: a wind tunnel study of a long-distance migrant shorebird. J Exp Biol 205: 2453–2460. doi:10.1145/2994205.2994212.

34. Guglielmo CG (2018) Obese super athletes: fat-fueled migration in birds and bats. J Exp Biol 221: jeb165753. doi:10.1242/jeb.165753.

35. Klaiman JM, Price ER, Guglielmo CG (2009) Fatty acid composition of pectoralis muscle membrane, intramuscular fat stores and adipose tissue of migrant and wintering white-throated sparrows (*Zonotrichia albicollis*). J Exp Biol 212: 3865–3872. doi:10.1242/jeb.034967.

36. McWilliams SR, Guglielmo C, Pierce B, Klaassen M (2004) Flying, fasting, and feeding in birds during migration: a nutritional and physiological ecology perspective. Source J Avian Biol 35: 377–393.

37. Ramenofsky M (1990) Fat storage and fat metabolism in relation to migration. In: Gwinner E, editor. Bird Migration Physiology and Ecophysiology. Berlin Heidelberg: Springer-Verlag. pp. 214–231. doi:10.1007/978-3-642-74542-3.

38. Parrish JD (1997) Patterns of frugivory and energetic condition in Nearctic landbirds during autumn migration. Condor 99: 681–697. doi:10.2307/1370480.

39. Catoni C, Metzger B, Schaefer HM, Bairlein F (2011) Garden Warbler, *Sylvia borin*, detect carotenoids in food but differ strongly in individual food choice. J Ornithol 152: 153–159. doi:10.1007/s10336-010-0560-6.

40. Catoni C, Peters A, Schaefer HM (2009) Dietary flavonoids enhance conspicuousness of a melanin-based trait in male blackcaps but not of the female homologous trait or of

sexually monochromatic traits. J Evol Biol 22: 1649–1657. doi:10.1111/j.1420-9101.2009.01778.x.

41. Costantini D, Cardinale M, Carere C (2007) Oxidative damage and anti-oxidant capacity in two migratory bird species at a stop-over site. Comp Biochem Physiol - C Toxicol Pharmacol 144: 363–371. doi:10.1016/j.cbpc.2006.11.005.

42. Gavino VC, Miller JS, Ikharebha SO, Milo GE, Cornwell DG (1981) Effect of polyunsaturated fatty acids and antioxidants on lipid peroxidation in tissue cultures. J Lipid Res 22: 763–769.

43. Tatner P, Bryant DM (1986) Flight cost of a small passerine measured using doubly labeled water: implications for energetics studies. Auk 103: 169–180.

44. Wikelski M, Tarlow EM, Raim A, Diehl RH, Larkin RP, et al. (2003) Costs of migration in free-flying songbirds. Nature 423: 704.

45. Jenni-Eiermann S, Jenni L (1991) Metabolic responses to flight and fasting in night-migrating passerines. J Comp Physiol B 161: 465–474. doi:10.1007/BF00257901.

46. Guglielmo CG (2010) Move that fatty acid: fuel selection and transport in migratory birds and bats. Integr Comp Biol 50: 336–345. doi:10.1093/icb/icq097.

47. Maillet D, Weber J-M (2007) Relationship between n-3 PUFA content and energy metabolism in the flight muscles of a migrating shorebird: evidence for natural doping. J Exp Biol 210: 413–420. doi:10.1242/jeb.02660.

48. Pierce BJ, McWilliams SR, Place AR, Huguenin MA (2004) Diet preferences for specific fatty acids and their effect on composition of fat reserves in migratory Red-eyed Vireos (Vireo olivaceous). Comp Biochem Physiol - A Mol Integr Physiol 138: 503–514. doi:10.1016/j.cbpb.2004.06.014.

49. Smith SB, McWilliams SR (2010) Patterns of fuel use and storage in migrating passerines in relation to fruit resources at autumn stopover site. Auk 127: 108–118. doi:10.1525/auk.2009.09139.

50. Place AR, Stiles EW (1992) Living off the wax of the land: bayberries and yellow-rumped warblers. Auk 109: 334–345. doi:10.2307/4088202.

51. Costantini D (2019) Understanding diversity in oxidative status and oxidative stress: the opportunities and challenges ahead. J Exp Biol 222: jeb194688. doi:10.1242/jeb.194688.

52. Cohen A, Klasing K, Ricklefs R (2007) Measuring circulating antioxidants in wild birds. Comp Biochem Physiol - B Biochem Mol Biol 147: 110–121. doi:10.1016/j.cbpb.2006.12.015.

53. Veskoukis AS, Nikolaidis MG, Kyparos A, Kouretas D (2009) Blood reflects tissue oxidative stress depending on biomarker and tissue studied. Free Radic Biol Med 47: 1371–1374. doi:10.1016/j.freeradbiomed.2009.07.014.

54. Cooper-Mullin C, Carter WA, McWilliams SR (2019) Acute effects of intense exercise on the antioxidant system in birds: does exercise training help? J Exp Biol 222: jeb.210443. doi:10.1242/jeb.210443.

55. Jimenez AG (2018) “The same thing that makes you live can kill you in the end”: exploring the effects of growth rates and longevity on cellular metabolic rates and oxidative stress in mammals and birds. Integr Comp Biol: 1–15. doi:10.1093/icb/icy090.

56. Catoni C, Peters A, Martin Schaefer H (2008) Life history trade-offs are influenced by the diversity, availability and interactions of dietary antioxidants. Anim Behav 76: 1107–1119. doi:10.1016/j.anbehav.2008.05.027.

57. Galli F, Azzi A, Birringer M, Cook-Mills JM, Eggersdorfer M, et al. (2017) Vitamin E:

emerging aspects and new directions. Free Radic Biol Med 102: 16–36. doi:10.1016/j.freeradbiomed.2016.09.017.

58. Traber MG, Atkinson J (2007) Vitamin E, antioxidant and nothing more. Free Radic Biol Med 43: 4–15. doi:10.1016/j.freeradbiomed.2007.03.024.

59. Surai PF, Sparks NHC, Acamovic T, McDevitt RM (2002) Antioxidant systems in the developing chicken: vitamins E and C in the liver of broiler chicks. Br Poult Sci 43: 64–65. doi:10.1080/000716602762388770.

60. Mazur-Kuśnirek M, Antoszkiewicz Z, Lipiński K, Kaliniewicz J, Kotlarczyk S, et al. (2019) The effect of polyphenols and vitamin E on the antioxidant status and meat quality of broiler chickens exposed to high temperature. Arch Anim Nutr 00: 1–16. doi:10.1080/1745039X.2019.1572342.

61. Van Kempen TATG, Reijersen MH, De Bruijn C, De Smet S, Michiels J, et al. (2016) Vitamin E plasma kinetics in swine show low bioavailability and short half-life of all-rac-α-tocopheryl acetate. J Anim Sci 94: 4188–4195. doi:10.2527/jas.2016-0640.

62. Arnold RN (1993) Tissue equilibration and subcellular distribution of vitamin E relative to myoglobin and lipid oxidation in displayed beef. J Anim Sci 71: 105–118.

63. Lauridsen C, Jensen SK (2012) α-Tocopherol incorporation in mitochondria and microsomes upon supranutritional vitamin E supplementation. Genes Nutr 7: 475–482. doi:10.1007/s12263-012-0286-6.

64. Lauridsen C, Engel H, Jensen SK, Craig a M, Traber MG (2002) Lactating sows and suckling piglets preferentially incorporate RRR- over all-rac-alpha-tocopherol into milk, plasma and tissues. J Nutr 132: 1258–1264.

65. Fariss MW, Nicholls-Grzemski FA, Tirmenstein MA, Zhang JG (2001) Enhanced

antioxidant and cytoprotective abilities of vitamin E succinate is associated with a rapid uptake advantage in rat hepatocytes and mitochondria. Free Radic Biol Med 31: 530–541. doi:10.1016/S0891-5849(01)00615-3.

66. Hacquebard M, Carpentier YA (2005) Vitamin E: absorption, plasma transport and cell uptake. Curr Opin Clin Nutr Metab Care 8: 133–138. doi:10.1097/00075197-200503000-00005.

67. Nudds RL, Bryant DM (2001) Exercise training lowers the resting metabolic rate of zebra finches, *Taeniopygia guttata*. Funct Ecol 15: 458–464. doi:10.1046/j.0269-8463.2001.00546.x.

68. Weber TP, Piersma T (1996) Basal metabolic rate and the mass of tissues differing in metabolic scope: migration-related covariation between individual knots *Calidris canutus*. J Avian Biol 27: 215. doi:10.2307/3677225.

69. Blatt DH, Leonard SW, Traber MG (2001) Vitamin E kinetics and the function of tocopherol regulatory proteins. Nutrition 17: 799–805. doi:10.1016/S0899-9007(01)00637-2.

70. Bruno RS, Leonard SW, Park S Il, Zhao Y, Traber MG (2006) Human vitamin E requirements assessed with the use of apples fortified with deuterium-labeled α-tocopheryl acetate. Am J Clin Nutr 83: 299–304. doi:10.1121/1.1907169.

71. Carter WA, Cooper-Mullin C, McWilliams SR (2018) Turnover of muscle lipids and response to exercise differs between neutral and polar fractions in a model songbird, the Zebra Finch. J Exp Biol 221: jeb168823. doi:10.1242/jeb.168823.

72. Ademowo OS, Dias HKI, Burton DGA, Griffiths HR (2017) Lipid (per) oxidation in mitochondria: an emerging target in the ageing process? Biogerontology 18: 859–879.

doi:10.1007/s10522-017-9710-z.

73. Jimenez AG, O’Connor ES, Elliott KH (2019) Muscle myonuclear domain, but not oxidative stress, decreases with age in a long-lived seabird with high activity costs. J Exp Biol: jeb.211185. doi:10.1242/jeb.211185.

74. Isaksson C (2013) Opposing effects on glutathione and reactive oxygen metabolites of sex, habitat, and spring date, but no effect of increased breeding density in great tits (*Parus major*). Ecol Evol 3: 2730–2738. doi:10.1002/ece3.663.

75. Giordano M, Costantini D, Tschirren B (2015) Sex-specific effects of prenatal and postnatal nutritional conditions on the oxidative status of great tit nestlings. Oecologia 177: 123–131. doi:10.1007/s00442-014-3100-x.

76. Roager HM, Sulek K, Skov K, Frandsen HL, Smedsgaard J, et al. (2014) *Lactobacillus acidophilus* NCFM affects vitamin E acetate metabolism and intestinal bile acid signature in monocolonized mice. Gut Microbes 5: 296–303. doi:10.4161/gmic.28806.

77. Carey H V., Duddleston KN (2014) Animal-microbial symbioses in changing environments. J Therm Biol 44: 78–84. doi:10.1016/j.jtherbio.2014.02.015.

78. Kono N, Arai H (2015) Intracellular Transport of Fat-Soluble Vitamins A and E. Traffic 16: 19–34. doi:10.1111/tra.12231.

79. Skrip MM, Seeram NP, Yuan T, Ma H, McWilliams SR (2016) Dietary antioxidants and flight exercise in female birds affect allocation of nutrients to eggs: how carry-over effects work. J Exp Biol 219: 2716–2725. doi:10.1242/jeb.137802.

80. Castiglione GM, Xu Z, Zhou L, Duh EJ (2020) Adapatation of the master antioxidant response connects metabolism, lifespan and feather development pathways in birds. Nat Commun 11: 1–15. doi:10.1038/s41467-020-16129-4.

81. Rosenberg K V, Dokter AM, Blancher PJ, Sauer JR, Smith AC, et al. (2019) Decline of the North American avifauna. Science (80- ) 124: 120–124.

82. Podsedek A (2007) Natural antioxidants and antioxidant capacity of Brassica vegetables: a review. LWT - Food Sci Technol 40: 1–11. doi:10.1016/j.lwt.2005.07.023.

83. Bauchinger U, McWilliams SR (2010) Carbon turnover in tissues of a passerine bird: Allometry, isotopic clocks, and phenotypic flexibility in organ size. Physiol Biochem Zool 83: 1032–1032. doi:10.1086/657659.

84. Carter WA, Whiteman JP, Cooper-Mullin C, Newsome SD, McWilliams SR (2019) Dynamics of individual fatty acids in muscle fat stores and membranes of a songbird and its functional and ecological importance. Physiol Biochem Zool 92: 239–251. doi:10.1086/702667.

85. Ristow M, Zarse K, Oberbach A, Klöting N, Birringer M, et al. (2009) Antioxidants prevent health-promoting effects of physical exercise in humans. PNAS 2: 2–7. doi:10.1073 pnas.0903485106.

86. Speakman JR (1998) The history and theory of the doubly labeled water technique. Am J Clin Nutr 68: 932–938. doi:10.1093/ajcn/68.4.932S.

87. Król E, Speakman JR (1999) Isotope dilution spaces of mice injected simultaneously with deuterium, tritium and oxygen-18. J Exp Biol 202: 2839–2849.

88. Scott GR, Egginton S, Richards JG, Milsom WK (2009) Evolution of muscle phenotype for extreme high altitude flight in the bar-headed goose. Proc R Soc B Biol Sci 276: 3645–3653. doi:10.1098/rspb.2009.0947.

89. Armstrong C, Thomas RH, Price ER, Guglielmo CG, Staples JF (2011) Remodeling mitochondrial membranes during arousal from hibernation. Physiol Biochem Zool 84:

438–449. doi:10.1086/660892.

90. Frezza C, Cipolat S, Scorrano L (2007) Organelle isolation: functional mitochondria from mouse liver, muscle and cultured filroblasts. Nat Protoc 2: 287–295. doi:10.1038/nprot.2006.478.

91. Gerson AR, Brown JCL, Thomas R, Bernards MA, Staples JF (2008) Effects of dietary polyunsaturated fatty acids on mitochondrial metabolism in mammalian hibernation. J Exp Biol 211: 2689–2699. doi:10.2174/138161209789909692.

92. Burnham KP, Anderson D. R. (2002) Model Selection and Multimodel Inference. Second. New York: Springer-Verlag. doi:10.1016/j.ecolmodel.2003.11.004.

93. Chou CC, Sung YC, Davison G, Chen CY, Liao YH (2018) Short-term high-dose vitamin C and E supplementation attenuates muscle damage and inflammatory responses to repeated taekwondo competitions: A randomized placebo-controlled trial. Int J Med Sci 15: 1217–1226. doi:10.7150/ijms.26340.

94. Costantini D (2014) Oxidative stress and hormesis in evolutionary ecology and physiology: A marriage between mechanistic and evolutionary approaches. Heidelberg, New York, Dordrecht, London: Springer-Verlag. 1–348 p. doi:10.1007/978-3-642-54663-1.

95. Lauridsen C, Buckley DJ, Morrissey PA (1997) Influence of dietary fat and vitamin E supplementation on α-tocopherol levels and fatty acid profiles in chicken muscle membranal fractions and on susceptibility to lipid peroxidation. Meat Sci 46: 9–22. doi:10.1016/S0309-1740(97)00010-7.

96. Tappel AL, Zalkin H (1959) Inhibition of lipide peroxidation in mitochondria by vitamin E. Arch Biochem Biophys 80: 333–336. doi:10.1016/0003-9861(59)90259-0.

97. Atkinson J, Harroun T, Wassall SR, Stillwell W, Katsaras J (2010) The location and behavior of α-tocopherol in membranes. Mol Nutr Food Res 54: 641–651. doi:10.1002/mnfr.200900439.

98. Burton GW, Traber MG (2003) Vitamin E: Antioxidant Activity, Biokinetics, and Bioavailability. Annu Rev Nutr 10: 357–382. doi:10.1146/annurev.nu.10.070190.002041.

99. Niki E, Abe K (2019) Vitamin E: Structure, Properties and Functions. In: Niki E, editor. Food Chemistry, Function and Analysis No.11. The Royal Society of Chemistry. pp. 1–11. doi:10.1039/9781788016216-00001.

100. Valko M, Leibfritz D, Moncol J, Cronin MTD, Mazur M, et al. (2007) Free radicals and antioxidants in normal physiological functions and human disease. Int J Biochem Cell Biol 39: 44–84. doi:10.1016/j.biocel.2006.07.001.

101. Leonard SW, Good CK, Gugger ET, Traber MG (2004) Vitamin E bioavailability from fortified breakfast cereal is greater than that from encapsulated supplements. Am J Clin Nutr 79: 86–92. doi:10.1093/ajcn/79.1.86.

102. Traber MG, Leonard SW, Bobe G, Fu X, Saltzman E, et al. (2015) α-tocopherol disappearance rates from plasma depend on lipid concentrations: Studies using deuterium-labeled collard greens in younger and older adults. Am J Clin Nutr 101: 752–759. doi:10.3945/ajcn.114.100966.

103. Jeanes YM, Hall WL, Lodge JK (2005) Comparative 2H-labelled α-tocopherol biokinetics in plasma, lipoproteins, erythrocytes, platelets and lymphocytes in normolipidaemic males. Br J Nutr 94: 92. doi:10.1079/BJN20051434.

104. Corder KR, DeMoranville KJ, Russell DE, Huss JM, Schaeffer PJ (2016) Annual life-stage regulation of lipid metabolism and storage and association with PPARs in a migrant

species: the gray catbird ( *Dumetella carolinensis* ). J Exp Biol 219: 3391–3398. doi:10.1242/jeb.141408.

105. DeMoranville KJ, Corder KR, Hamilton A, Russell DE, Huss JM, et al. (2019) PPAR expression, muscle size, and metabolic rates across the Gray catbird's annual cycle are greatest in preparation for fall migration. J Exp Biol 45056: jeb.198028. doi:10.1242/jeb.209452.

**S1 Table Caption:**

**Model Selection to explain δD-α-tocopherol values in whole blood and mitochondria**

Number of model parameters (K), maximized log-likelihood [log(L)], change in second-order Akaike Information Criterion for small sample sizes (AICc), and AICc weights (wi) for each of the candidate models used to determine if the amount of δD-α-tocopherol in a tissue varied with treatment group (Trained, Untrained) as referenced by background deuterium levels, the time that a bird was gavaged (22.5 hrs or 6.5 hrs prior to sampling), and the amount of administered δD α-tocopherol (mL olive oil mixture). Based on estimated effect sizes, the power to detect changes for blood and mitochondria was 0.80 and 0.98, respectively. Separate models were run for each tissue measured.

Figure 1 Click here to access/download;Figure;Fig1_plosone.png

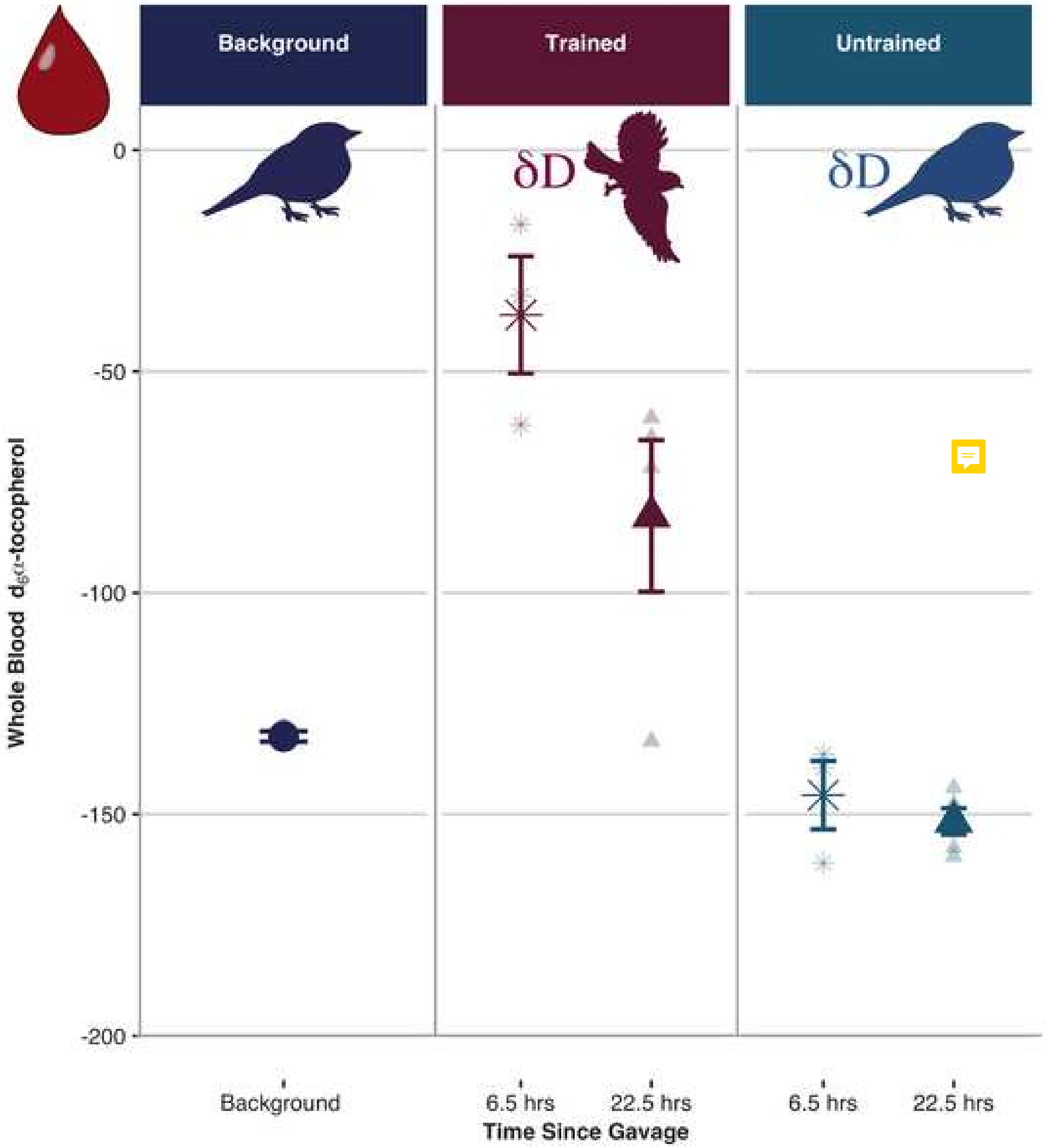

Figure 2

Click here to access/download;Figure;Fig2_plosone.png

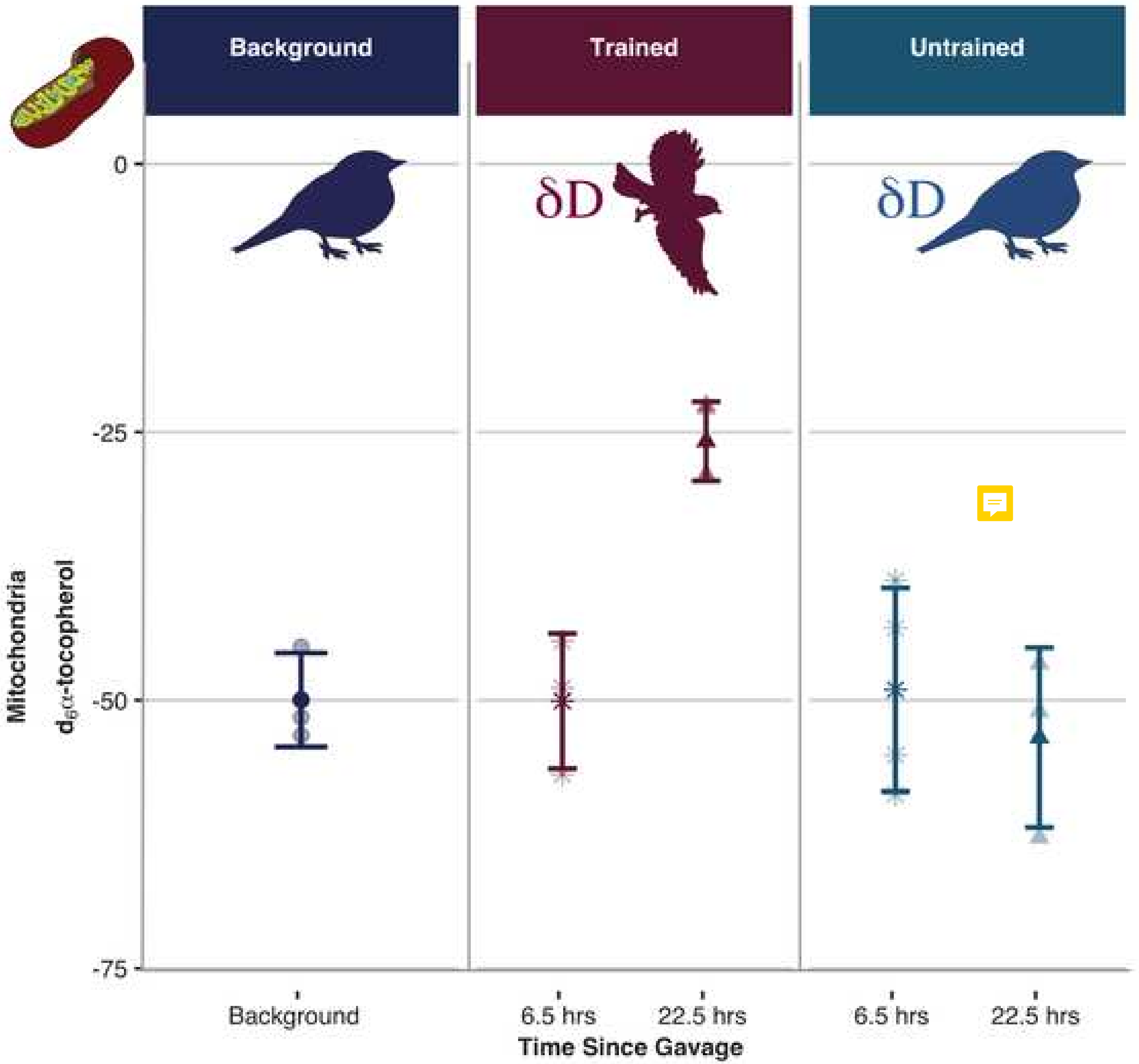

Click here to access/download
**Supporting Information**
SuppInfo_PLOSone.docx

R Code #1

Click here to access/download
**Supporting Information**
ESM_Rcode1.R

R Code #2

Click here to access/download
**Supporting Information**
ESM_Rcode2.R

Data

Click here to access/download
**Supporting Information**
ZEFI_isotope3.csv
