# Supplementary material for: Dietary vitamin E reaches the mitochondria in the flight muscle of zebra finches but only if they exercise
Source: PLoS One. 2021 Jun 28;16(6):e0253264. doi: 10.1371/journal.pone.0253264 (PMC8238215; doi:10.1371/journal.pone.0253264)
Supplement: Attachment — Submitted filename: Rebuttal Letter 2 for PONE.docx [file pone.0253264.s006.docx]

**Rebuttal Letter (#2) for PONE-D-20-29149: Dietary vitamin E reaches the mitochondria in the flight muscle of zebra finches but only if they exercise**

**Reviewer #2:**

*I commend the authors for making many improvements to the manuscript since the previous round of revisions. While the manuscript is overall good, there are still some areas to address before it is suitable for publication. There are points in the main text of the manuscript that require additional clarification, explanation and consistency. In addition, the figures require additional attention, to reduce confusion and difficulty in interpretation by the reader. Please see my specific comments on the manuscript for additional details.*

**Thank you for your careful review of our manuscript. We have thoroughly thought through your comments and responded below. Below in bold are our responses to each comment and suggestion for revision. The line numbers associated with your comments are associated with the previous unmarked version of the manuscript and the line numbers associated with specific changes to our manuscript refer to the current revised unmarked version of our paper without tracked changes.**

*Line 43: “Mitochondria are the key sites of energy metabolism in all air breathing organisms.”*

- *While true, this statement could be expanded to be more accurate. Fish do not breathe air yet their mitochondria are key sites of energy metabolism.*

**We thank the reviewer for this comment and have changed the sentence to read (Line 43):**

**“Mitochondria are the key sites of energy metabolism in all oxygen consuming organisms.”**

*Line 58: “Although the functional importance of dietary antioxidants to animal health and performance is recognized [16–18], no previous studies have demonstrated that consumed antioxidants are transported to the primary site of reactive species production, the mitochondria, and whether this depends at all on the physiological state of the animal.”*

- *Some emerging evidence has indicated that mitochondria may not be as significant of a reactive species production as previously thought. Please see: Zhang, Y., & Wong, H. S. (2021). Are mitochondria the main contributor of reactive oxygen species in cells?. Journal of Experimental Biology, 224(5), jeb221606.*

**We thank the reviewer for pointing out this recent commentary and have changed this sentence to read (Line 56):**

**“Although the functional importance of dietary antioxidants to animal health and performance is recognized [16–18], no previous studies have demonstrated that consumed antioxidants are transported to a major site of RS production, the mitochondria (but see: [19]), and whether this depends at all on the physiological state of the animal.”**

*Line 64: “Flight is a particularly energetically expensive form of exercise and is primarily fueled by catabolizing stored fat - nature’s most energy dense, yet oxidatively vulnerable fuel [27,28].”*

- *Specify here that you are referring to "metabolic" fuels.*

**We thank the reviewer for this comment and have clarified that we are referring to metabolic fuels (Line 64):**

**“Flight is a particularly energetically expensive form of exercise and is primarily fueled by catabolizing stored fat - nature’s most energy dense, yet oxidatively vulnerable metabolic fuel [28,29].”**

*Line 81: “Importantly, if and only if dietary antioxidants are absorbed and then transported to the mitochondria, can they effectively alleviate the oxidative costs associated with energy-demanding life events such as flying.”*

- *"assimilated by the gut" would be a preferable terminology to describe this process.*

**We thank the reviewer for this comment and have changed the sentence accordingly (Line 81):**

**“Importantly, if and only if dietary antioxidants are assimilated by the gut and then transported to the mitochondria, can they effectively alleviate the oxidative costs associated with energy-demanding life events such as flying.”**

*Line 103: “reactive species”*

- *Reactive species were defined as "RS" on line 44; stay consistent with acronyms.*

**We thank the reviewer for catching this oversight and have changed all mentions of reactive species after line 44 to RS.**

*Lies 157-160: “We sampled only males because a previous study found differences in oxidative damage associated with flight-training between males and females [28]. Due to time constraints associated with extracting mitochondria while keeping tissues alive (see below), we sacrificed subsets of males in each treatment group at two different time points as follows.”*

- *This wording is unclear; is the authors' point that the sampling regime was chosen due to decline in mitochondrial quality with time?*

**Thank you for asking us to explain our methods. In order to properly isolate mitochondria from muscle, the tissue must be kept on ice and “alive” throughout the entire isolation procedure. Therefore, it was impossible for us to process more than 12 birds in a given day. We have changed our wording to clarify this issue (Line 160):**

**“Due to time constraints associated with the need to keep the tissues cold and alive during the process of isolating mitochondria from pectoral muscle (see below), we sacrificed subsets of males in each treatment group at two different time points as follows.”**

*Lines 161 -165: “After 48 days of daily exercise training, we randomly selected 8 male zebra finches from the untrained group and 4 male zebra finches from the trained group to be gavaged with 150 μL (or 0.200 μmoles) of deuterated α – tocopherol (d6-RRR- α – tocopherol acetate, a gift from Dr. John Lodge, St. Thomas’ Hospital, London, hereafter: δD α-tocopherol or d6α-tocopherol) dissolved in olive oil.”*

- *Please be consistent here with your terminology; the next mention of the labelled tocopherol on line 165 does not follow either of these short-hand terms.*

**Thank you for making sure we are consistent. We have made edits where appropriate to make sure that we are consistent. Additionally, we expanded on our rationale for why we use two different terms (Lines 162-167:**

**“After 48 days of daily exercise-training, we randomly selected 8 male zebra finches from the unexercised group and 4 male zebra finches from the exercised group to be gavaged with 150 μL (or 0.200 μmoles) of deuterated α – tocopherol (d6-RRR- α – tocopherol acetate, a gift from Dr. John Lodge, St. Thomas’ Hospital, London, hereafter: d6α-tocopherol or δD α-tocopherol to indicate the isotopic ratio) dissolved in olive oil.”**

*Line 168: “150 μL of deuterated α – tocopherol (δD α-tocopherol)”*

- *Unclear why this is defined again after being defined on lines 164-165.*

**We thank the reviewer for this comment and have changed this sentence in the manuscript (Line 169):**

**“After 70 days of exercise-training, we randomly selected a second group of 4 male zebra finches from the exercised group to be gavaged with 150 μL of d6α-tocopherol, and 3 male zebra finches from the unexercised group to be gavaged with 150 μL of olive oil.”**

*Line 177: “in vivo”*

- *This should be italicized*

**We thank the reviewer for this observation and have corrected this issue in the manuscript.**

*Line 179: “took a blood sample from the severed carotid artery”*

- *Avian red blood cells contain mitochondria that presumably generate reactive species and likely contain antioxidants, such as a-tocopherol. How much of the a-tocopherol detected is in the VLDLs and hence available to be taken up by systemic tissues?*

**We entirely agree with the reviewer that understanding the amount of α-tocopherol circulating in VLDLs would be interesting. However, we did not isolate VLDLs from the whole blood to measure the amount of labeled α-tocopherol and therefore we cannot address this issue. That being said, the fact that the labeled α-tocopherol was found in the isolated mitochondria from pectoral muscle indicates that at least some of the ingested α-tocopherol was available to be taken up by tissues (at least in birds that experienced exercise).**

*Line 182: “Mitochondria from each bird were isolated using differential centrifugation and a Percoll gradient [67–70].”*

- *Please specify here that you isolated mitochondria from the pectoralis*

**We thank the reviewer for this comment and have clarified this in the manuscript (Line 184):**

**“Mitochondria from the pectoral muscle of each bird were isolated using differential centrifugation and a Percoll gradient [67–70].”**

*Line 284 (Figure 1 Legend): “Figure 1. Whole blood δD-α-tocopherol values from background (dark blue circle), exercised (red) and unexercised (light blue) Zebra Finches.”*

- *While these values were explained in the main text, please provide a brief explanation on the nature of these values in the figure caption. Figures and their associated captions should be interpreted independently of the main text. Please repeat for Fig. 2.*

**We have added an explanation of the values to the figure legends (see our response to the comment below).**

- *Please be consistent with terminology; it is unclear why zebra finch is capitalized in this case and in Fig. 2, yet is not capitalized in the main text.*

**Thank you for keeping our language and terminology consistent. We have changed it so all mentions of zebra finches are not capitalized throughout the manuscript.**

*Line 289: “Deuterium values in the blood of untrained birds were not different from background at either sampling time point”*

- *Similar to a previous comment on figure captions, please provide a brief explanation of what "background" means. Please repeat for Fig. 2*

**Thank you for making sure our figures are clear. We have updated the figure captions to be standalone:**

**“Fig 1. Whole blood δD-α-tocopherol values from background (dark blue circle), exercised (red) and unexercised (light blue) zebra finches.**

**Only exercise-trained birds had deuterium values (δD α-tocopherol) that were higher than background, and exercise-trained birds gavaged 6.5 hours prior to sampling (red star) had higher deuterium values than birds gavaged 22.5 hours prior to sampling (red triangle). Background samples were obtained from birds that were gavaged with olive oil only. Exercised and unexercised samples were obtained from birds gavaged with d6α-tocopherol (indicated by δD). The stable isotope ratios are reported in delta-notation as parts per thousand (‰) deviations from the international standard for deuterium (δD). δD α-tocopherol values in the blood of unexercised birds were not different from background at either sampling time point (gavaged 6.5 hours prior to sampling = light blue star; gavaged 22.5 hours prior to sampling = light blue triangle; adjusted R2 = 0.79, Exercised: ß = 93.45, t3,14 = 5.03, P < 0.001; Unexercised: ß = 8.59, t3,14 = 0.46, P = 0.65; Hours Since Gavage: ß = -1.55, t3,14 = -2.18, P = 0.05). Lighter colored points represent the raw data, points and error bars represent mean ± SE.”**

**“Fig 2. δD α-tocopherol values of mitochondria isolated from the pectoral muscles of background (dark blue circle), exercised (red) and unexercised (light blue) zebra finches.**

**Only exercise-trained birds had deuterium values that were higher than background, and exercise-trained birds gavaged 6.5 hours prior to sampling (red star) had lower deuterium values than birds gavaged 22.5 hours prior to sampling (red triangle). Background samples were obtained from birds that were gavaged with olive oil only. Exercised and unexercised samples were obtained from birds gavaged with d6α-tocopherol (indicated by δD). The stable isotope ratios are reported in delta-notation as parts per thousand (‰) deviations from the international standard for deuterium (δD). δD α-tocopherol values in the blood of unexercised birds were not different from background at either sampling timepoint (gavaged 6.5 hours prior to sampling = light blue star; gavaged 22.5 hours prior to sampling = light blue triangle; adjusted R2 = 0.70, Interaction: ß = 1.80, t3,10 = 3.65, P = 0.004; Exercised: ß = -12.73, t3,10 = -1.60, P = 0.15; Hours Since Gavage: ß = -0.28, t3,10 = -0.80, P = 0.44). Lighter colored points represent the raw data, points and error bars represent mean ± SE.”**

*Line 310: “mitochondria of zebra finches”*

- *Specify here that you are referring to pectoralis mitochondria.*

**We thank the reviewer for this comment and have clarified that we are referring to mitochondria from the pectoralis (Lines 319-321):**

**“Specifically, δD-α-tocopherol was found in the blood and mitochondria isolated from the pectoralis muscle of zebra finches within 6.5 and 22.5 hrs, respectively, but only if the birds were exercise-trained.”**

*Line 315: “the idea that increased RS production associated with exercise facilitated the absorption and deposition of α-tocopherol seems likely.”*

- *Please expand on this rationale: is there a mechanism that could explain how increased RS production facilitates dietary antioxidant assimilation? If so, please explain it.*

**Thank you for this comment. However, the focus of our paper was on the extent to which a dietary antioxidant consumed by birds was absorbed and deposited in muscle mitochondria and whether this depended on exercise. Given that our study was not designed to elucidate the mechanisms, we prefer to refrain from providing further conjecture about the mechanisms.**

*Line 324: “overwhelming damage”*

- *Somewhat hyperbolic; oxidative stress or damage would be more appropriate terminology*

**We thank the reviewer for this comment and have changed the sentence to read (Line 330):**

**“The absence of δD-α-tocopherol levels above baseline in unexercised birds suggests that any baseline RS generation or that associated with short flights within the cages could be neutralized via endogenous antioxidants (enzymes, sacrificial molecules, or stores of dietary antioxidants), or were in low enough of a dose to act solely as cellular messengers rather than cause oxidative stress [2].”**

*Line 330: “This residence time of α-tocopherol in the blood of trained zebra finches is more rapid than in plasma of humans given deuterium-labeled foods (half-life = ~ 30 hrs), and more rapid than uptake into human erythrocytes [82].”*

- *This statement requires some re-wording: 1) "residence time" is a duration, so it can be or "shorter" rather than "more rapid." 2) the term half-life here is confusing; I presume it refers to the food itself, not the deuterium (being a stable isotope) but the phrasing may confuse some readers.*

**Thank you for pushing us to make this manuscript as accessible as possible. We have changed the wording to “shorter” rather than “more rapid” to be consistent with a duration. Additionally, we previously define the term half life on Lines 280-283:**

**“Estimated retention time (τ) in whole blood converged on a value of 24.72 ± 12.76 by 180+ hrs after gavage and the median residence time, or half-life (ln(2) *τ), of gavaged δD α-tocopherol in whole blood of exercise-trained zebra finches was estimated at 17.1 hrs. In other words, 90% of gavaged δD-α-tocopherol that appeared in the blood would be replaced or deposited into tissues within 2.4 days.”**

*Line 356: “mechanisms associated with fatty acid oxidation”*

- *Do any of these mechanisms include increased fatty acid uptake? E.g. fatty acid translocase/CD36, FABP which may be more relevant to the uptake of a-tocopherol than an oxidizing enzyme like HOAD.*

**The reviewer is absolutely correct that fatty acid uptake is important for the uptake of tocopherol and some mechanisms associated with fatty acid uptake are also upregulated during migration. Therefore, we have added this information to the discussion (Lines 365-370):**

**“Birds rely primarily on fatty acid oxidation to fuel the demands of intense exercise and, in the wild, mechanisms associated with fatty acid transport (e.g. fatty acid translocase/CD36 or plasma membrane-bound fatty acid binding protein) and fatty acid oxidation (e.g. activation of PPARs, enzymes such as β-hydroxyacyl-Coenzyme-A dehydrogenase or carnitine palmitoyl transferase) are upregulated prior to seasonal migration [85–87].”**

*Figures:*

- *Provide some explanation as to what the "dD" symbol here and under Untrained represents. Repeat for Fig. 2*

**As above, we have updated the figure legends to explain all the symbols and terms.**

- *The y-axis title requires units. Repeat for Fig. 2*

**We thank the reviewer for this suggestion and have updated the figures to reflect the units as parts per thousand (*‰*) deviations from the international standard for deuterium.**

- *Unclear why this page is landscape when all others are portrait.*

**This page was automatically formatted by PLOS ONE. The figures can be downloaded in high resolution for best viewing by clicking the link in the corner.**

*Other: “Stable isotope ratios are reported in delta-notation as parts per thousand (‰)*

*deviations from the international standard for deuterium (δD).”*

- *This is a nice explanation that should be included in the figure captions*

**We are glad you think this is a good explanation and we have now included it in the figure captions.**
